# Molecular identification of *Cryptosporidium* spp. in seagulls, pigeons, dogs, and cats in Thailand

**DOI:** 10.1051/parasite/2014053

**Published:** 2014-10-10

**Authors:** Khuanchai Koompapong, Hirotake Mori, Nipa Thammasonthijarern, Rapeepun Prasertbun, Ai-rada Pintong, Supaluk Popruk, Wichit Rojekittikhun, Kittipong Chaisiri, Yaowalark Sukthana, Aongart Mahittikorn

**Affiliations:** 1 Department of Protozoology, Faculty of Tropical Medicine, Mahidol University Bangkok Thailand; 2 Department of Tropical Pediatrics, Faculty of Tropical Medicine, Mahidol University Bangkok Thailand; 3 Department of Helminthology, Faculty of Tropical Medicine, Mahidol University Bangkok Thailand

**Keywords:** *Cryptosporidium*, pigeon, seagull, dog, cat, Thailand

## Abstract

Zoonotic *Cryptosporidium* spp., particularly *C. meleagridis*, *C. canis*, and *C. felis*, are enteric protozoa responsible for major public health concerns around the world. To determine the spread of this parasite in Thailand, we conducted molecular identification of *Cryptosporidium* spp. from animal samples around the country, by collecting and investigating the feces of seagulls (*Chroicocephalus brunnicephalus* and *Chroicocephalus ridibundus*), domestic pigeons (*Columba livia domestica*), dogs, and cats. Seagull and pigeon samples were collected at the seaside and on the riverside to evaluate their potential for waterborne transmission. Ten pigeon samples were combined into one set, and a total of seven sets were collected. Seventy seagull samples were combined into one set, and a total of 13 sets were collected. In addition, 111 dog samples were collected from cattle farms, and 95 dog and 80 cat samples were collected from a temple. We identified *C. meleagridis* in pigeons, *Cryptosporidium* avian genotype III in seagulls, *C. canis* in dogs, and *C. felis* in cats. In the temple, the prevalence was 2.1% (2/95) for dogs and 2.5% (2/80) for cats. No *Cryptosporidium* was found in dog samples from cattle farms. These are the first findings of *C. meleagridis* in domestic pigeons, and *Cryptosporidium* avian genotype III in seagulls. Our study invites further molecular epidemiological investigations of C*ryptosporidium* in these animals and their environment to evaluate the public health risk in Thailand.

## Abstract (fr)

Les espèces zoonotique de *Cryptosporidium*, particulièrement *C. meleagridis*, *C. canis* et *C. felis*, sont des protozoaires entériques responsables de préoccupations majeures de santé publique à travers le monde. Pour la détermination de la propagation de ce parasite en Thaïlande, nous avons effectué l’identification moléculaire de *Cryptosporidium* spp. à partir d’échantillons d’animaux de tout le pays, en recueillant et en examinant les excréments de mouettes (*Chroicocephalus brunnicephalus* et *Chroicocephalus ridibundus*), pigeons domestiques (*Columba livia domestica*), chiens et chats. Les échantillons provenant de mouettes et de pigeons ont été recueillis en bord de mer ou de rivière pour évaluer leur potentiel de transmission par les eaux. Dix échantillons de pigeons ont été combinés en un seul ensemble, et un total de sept ensembles ont été collectées. Soixante-dix échantillons de mouettes ont été combinés en un seul ensemble, et un total de 13 ensembles ont été collectés. En outre, 111 échantillons de chiens ont été recueillis dans des fermes de bétail, et 95 échantillons de chiens et 80 échantillons de chats ont été recueillis d’un temple. Nous avons identifié *C. meleagridis* chez les pigeons, *Cryptosporidium* génotype aviaire III chez les mouettes, *C. canis* chez les chiens, et *C. felis* chez les chats. Dans le temple, la prévalence était de 2.1 % (2/95) pour les chiens et 2.5 % (2/80) pour les chats. *Cryptosporidium* n’a pas été trouvé dans les échantillons de chiens de fermes de bovins. Ceci est la première mention de *C. meleagridis* chez les pigeons domestiques, et de *Cryptosporidium* génotype III aviaire chez les mouettes. Notre étude montre la nécessité d’autres enquêtes épidémiologiques moléculaire sur *Cryptosporidium* chez ces animaux et leur environnement pour évaluer le risque pour la santé publique en Thaïlande.

## Introduction

*Cryptosporidium*, a common intestinal parasite of humans and animals, has a diverse global distribution and 30 species with >50 genotypes have been identified [47, 60]. In Europe, *C. parvum* and *C. hominis* are responsible for >95% of human cryptosporidiosis [9, 25, 51]. In the USA, Australia, and Japan, *C. hominis* is more prevalent than *C. parvum* [29, 59, 62]. *C. meleagridis*, *C. felis*, and *C. canis*, predominant species in birds, cats, and dogs, respectively, are generally less common than *C. hominis* and *C. parvum* [60]. In Thailand, cryptosporidiosis prevalence rates between 2.5 and 25% have been reported in HIV-infected patients in urban areas [53]. Particularly, *C. meleagridis*, *C. felis*, *and C. canis* have been found and are responsible for 35.2% of all *Cryptosporidium* infections in Thai HIV patients [11]. The risk of non-parvum zoonotic *Cryptosporidium* infection is considered high in Thailand, at least in immunodepressed patients.

To date, *Cryptosporidium* has been studied in dogs [3, 31], cattle [18, 19, 35], mussels [54], and chickens in Thailand, [20], but no molecular epidemiological investigations of *Cryptosporidium* in animals other than ruminants have been conducted. The main objective of our study was to identify *Cryptosporidium* in animals, especially focusing on birds, dogs, and cats, by a molecular method. We selected seagulls and domestic pigeons, common migratory and domestic birds that live near bodies of water, making them potential sources of water contamination. Many people visit seaside piers and riverside areas in Thailand for relaxation, and these areas often act as sanctuaries for birds since bird-feeding is a favored activity among both visitors and local people. In these areas, birds gather at precise times, waiting for people to feed them. They tend to be unafraid of human presence, and this proximity risks making these places important transmission zones for zoonotic diseases. For similar reasons, dogs in cattle farms, as well as dogs and cats in a temple were selected for investigation. Stray dogs and cats are often cared for at temples in Thailand due to religious beliefs, and temples have become known as endemic places for intestinal parasitic infections in animals living there [56].

## Materials and methods

### Study sites and sample collection

Seagull samples were collected at Bang Poo Nature Reserve Pier, in Samut Prakan Province, central Thailand. Most seagulls were brown-headed gulls (*Chroicocephalus brunnicephalus*) and black-headed gulls (*Chroicocephalus ridibundus*). These birds migrate from China in October–May each year, and the samples were collected in both 2010 and 2011. Due to the small amount of feces per dropping, fecal samples from 70 seagulls were combined into one set, and a total of 13 such sets were collected.

Samples from domestic pigeons (*Columba livia domestica*) were collected in September 2012 from three locations: (1) near the pier in Wat Rakang Kositaram; (2) Brahmin Swing; (3) the Pramane Ground. All locations are in Bangkok, central Thailand. Fecal samples from 10 pigeons were combined into one set, and a total of seven sets were collected. All bird samples were collected immediately when the feces dropped to the ground.

One hundred eleven dog samples were collected from 105 dairy cattle farms in central Thailand (Nakhon Pathom Province: Mueang Nakhon Pathom District; Ratchaburi Province: Photharam District, Ban Pong District; Lopburi Province: Phatthana Nikhom District; Sa Kaeo Province: Watthana Nakhon District, Mueang Sa Kaeo District, Wang Sombun District) in January–April 2012. Most of the dogs were adult. They appeared to be healthy and well fed by their owners. Dog samples were collected *per rectum* by experienced veterinarians. In addition, 95 samples from dogs and 80 from cats were collected from a temple in central Thailand (Ban Na District, Nakhon Nayok Province) in October 2012. About 1,000 dogs and 500 cats lived around the temple area, fed by monks and volunteers. Most of the dogs and cats were adult. Some animals were kept in cages, while others were free to roam. Dog and cat fecal samples were collected at random from the grounds. Samples from all animals were kept in dry tubes in cool conditions during transportation, and preserved at −20 °C for DNA extraction. This study was approved by the Animal Care and Use Committee, Faculty of Tropical Medicine, Mahidol University, Thailand.

### Molecular analysis

DNA was extracted from the samples using a commercially available DNA extraction kit (PSP Spin Stool DNA Kit, Stratec Inc., Germany) according to the manufacturer’s instructions. This kit contains optimized essential washing conditions to remove inhibitors very efficiently. Fragments of SSU rRNA (830 bp) were amplified by PCR, using primers and protocols described previously [61]. The PCR products were subjected to electrophoresis in 2% agarose gel and visualized by ethidium bromide staining. All amplified products were sequenced in both directions using the secondary PCR primers on an ABI 3730xl DNA Analyzer (Applied Biosystems). The *Cryptosporidium* species and genotypes from each specimen were confirmed by homology of the sequenced PCR products to the sequence published in GenBank.

## Results

The numbers of positive samples examined for *Cryptosporidium* for each collection site and the *Cryptosporidium* species/genotypes determined by PCR analysis of the SSU rRNA gene are summarized in Table 1. Two sets from seagulls, and one set from pigeons, were found positive for *Cryptosporidium* spp. with 100% homology with *Cryptosporidium* avian genotype III and *C. meleagridis* in the published sequences in GenBank (AB694729 and KF701463). *Cryptosporidium* was not identified in the dog samples from the cattle farms but 2/95 (2.1%) dog samples and 2/80 (2.5%) cat samples from the temple were found positive for *Cryptosporidium*. Genotyping analysis of the PCR-positive samples identified *C. canis* and *C. felis*, respectively, with 100% homology with the published sequences in GenBank (FJ233035 and JQ413437).

**Table 1.** Number of positive samples examined for *Cryptosporidium* for each collection site and the *Cryptosporidium* species/genotypes determined by PCR analysis of the SSU rRNA gene.

Type of animal	Collection site	Sample size	*Cryptosporidium*-positive (%)	*Cryptosporidium* species/genotype
Pigeons	Wat Rakang Kositaram, Bangkok	4 sets*	1 (25%)	*C. meleagridis*
	Brahmin Swing, Bangkok	2 sets*	0	
	The Pramane Ground, Bangkok	1 set*	0	
Seagulls	Bang Poo Nature Reserve Pier, Samut Prakan	13 sets**	2 (15.3%)	*C. avian genotype* III
Dogs	Nakhon Pathom	3	0	
	Ratchaburi	27	0	
	Lopburi	33	0	
	Sa Kaeo	48	0	
	Nakhon Nayok	95	2 (2.1%)	*C. canis*
Cats	Nakhon Nayok	80	2 (2.5%)	*C. felis*

* 1 set = 10 fecal samples;

** 1 set = 70 fecal samples.

## Discussion

Some *Cryptosporidium* species and genotypes are more associated with human illness than others, and some may be related to specific pathogenicity in human cryptosporidiosis [47]. Previously in Thailand, *Cryptosporidium* infections have been reported in food (12.5% in green mussels) [54], and the environment (12.7% in the waters of southwest coastal areas of Thailand) [52]. Unfortunately, species/genotype identification was not possible in previous studies in Thailand due to the use of the indirect fluorescent-antibody method and because no species-specific monoclonal antibodies were available. Otherwise, more detailed information on genotyping, particularly for human-adapted species, would allow more precise public health risk assessments for cryptosporidiosis in the future [53]. Similarly, our previous study using the nested PCR technique targeting the SSU rRNA gene revealed that 11% of river- and 6% of sea-water samples in central Thailand were contaminated with several *Cryptosporidium* species, including *C. parvum*, *C. meleagridis* and *C. serpentis*. The highest river contamination levels occurred during the rainy season, and the highest sea-water levels corresponded with the presence of migratory seagulls, indicating that runoff water carries the parasite into the rivers/sea [23]. Therefore, it is important to investigate the influence of domestic animals as potential transmission reservoirs for *Cryptosporidium*, and their impact on public health.

The domestic pigeon is one of the most common birds around the world. Herein, *C. meleagridis* was found for the first time in feces of domestic pigeons. To date, three avian *Cryptosporidium* species (*C. meleagridis*, *C. baileyi*, and *C. galli*) and 11 genotypes (avian genotypes I–V, black duck genotype, Eurasian woodcock genotype, and goose genotypes I–IV) have been reported [10, 58, 59]. Among them, *C. meleagridis* is pathogenic, causing diarrhea in both humans and birds [8, 41, 60]. As shown in Table 2, *C. meleagridis* has been identified globally in livestock, such as chickens, hens, and turkeys [2, 5, 13, 17, 33, 46, 48, 50, 55]. It has also been frequently reported in a variety of pet birds in Japan and China [1, 42]. A few studies have also found *C. meleagridis* in wild and domestic free-living birds [24, 30, 37].

**Table 2.** Avian host and country of identification of *C. meleagridis* and *Cryptosporidium* avian genotype III, from published records.

	Avian host	Country	References
*C. meleagridis*	Chicken and hen (*Gallus gallus domesticus*)	USA	[2, 5, 13, 17, 33, 46, 50]
		Sweden	
		Algeria	
		Brazil	
		Tunisia	
	Turkey (*Meleagris gallopavo*)	USA	[5, 48, 55]
		Italy	
		Algeria	
	Ring-necked parrot (*Psittacula krameri*)	Australia	[30]
	Cockatiel (*Nymphicus hollandicus*)	Japan	[1]
	Red-legged partridge (*Alectoris rufa*)	China	[42]
		Spain	[37]
	Rose-ringed parakeet (*Psittacula krameri*)		[42]
	Bohemian waxwing (*Bombycilla garrulus*)		[42]
	Rufous turtle dove (*Streptopelia orientalis*)		[42]
	Fan-tailed pigeon (*C. l. domestica*)		[42]
	Scale quail (*Callipepla squamata*)		[42]
	Domestic pigeon (*C. l. domestica*)	Thailand	This study
*Cryptosporidium* avian genotype III	Cockatiel (*N. hollandicus*)	Australia	[33, 34, 42]
		Brazil	
		China	
	Galah (*Eolophus roseicapilla*)	Australia	[34]
	Sun conure/parakeet (*Aratinga solstitialis*)	Australia	[34]
	Canada goose (*Branta canadensis*)	USA	[21]
	Red-billed blue magpie (*Urocissa erythrorhyncha*)	China	[42]
	Peach-faced lovebird (*Agapornis roseicollis*)	Brazil	[33]
	Brown-headed gull (*C. brunnicephalus*)	Thailand	This study
	Black-headed gull (*C. ridibundus*)		

Several recent studies report occurrences of *Cryptosporidium* in urban pigeons that are infectious to humans [15, 36, 42, 43]. Pigeons living in urban parks may be a source of zoonotic cryptosporidiosis, affecting primarily immunocompromised people, children, and the elderly [42]. We chose to collect domestic pigeon and seagull samples for this study in locations where many tourists gather to feed the birds. Our results indicate the need for further molecular epidemiological investigations of *Cryptosporidium* in domestic birds and their handlers, including pigeons, to evaluate the risk they pose to humans. *Cryptosporidium* are also well known for waterborne transmission as they are commonly found in wastewater [7, 24]. Our previous surveillance of river- and sea-water identified several *Cryptosporidium* species, including *C. parvum*, *C. meleagridis*, and *C. serpentis* [23]. The habitats of the pigeons in our study were very close to the pier and riverside, so they may cause water contamination.

*Cryptosporidium* avian genotype III was detected in seagull samples (*Chroicocephalus brunnicephalus* and *Chroicocephalus ridibundus*). Two previous studies have reported *Cryptosporidium* in seagulls, but this is the first study to identify *Cryptosporidium* genotypes in seagulls using a molecular method [40, 49]. *Cryptosporidium* avian genotype III has been detected in a variety of birds around the world (Table 2), such as the cockatiel (*Nymphicus hollandicus*), galah (*Eolophus roseicapilla*), sun conure/parakeet (*Aratinga solstitialis*), Canada goose (*Branta canadensis*), red-billed blue magpie (*Urocissa erythrorhyncha*), and peach-faced lovebird (*Agapornis roseicollis*) [21, 33, 34, 42], but has not been identified in livestock or other migratory birds. Because seagulls migrate long distances, they may be responsible for transmitting the parasite to other countries.

The prevalence of zoonotic *Cryptosporidium* spp. varies in different regions of the world. As shown in Table 3, in dogs, *C. canis* was the most prevalent species in Australia [39], *C. parvum* in Italy [12], and *C. muris* in the USA [26]. *Cryptosporidium* prevalence in dogs varies – high infection rates (>10%) have been reported in the USA and Norway [16, 26], with lower prevalence (<2%) in the UK, Brazil, and Australia [6, 32, 39]. *C. felis* is the predominant species in cats [4, 38, 45], while *Cryptosporidium* species tend to differ by study area. In cats, rates are generally higher than dogs; infection rates > 10% have been reported in Colombia, Italy, and the USA [4, 44, 45], and are reportedly low in the UK [14, 57]. In the present study, 2 out of 95 (2.1%) dog and 2 out of 80 (2.5%) cat fecal samples were positive for *Cryptosporidium.* The PCR technique, capable of detecting low levels of *Cryptosporidium* infection, was used in this study because *Cryptosporidium* can be difficult to detect using conventional microscopy, as found in previous reports [27, 28]. However, the prevalence of *Cryptosporidium* in dogs and cats was low. Given the low prevalence of this parasite found in our study, we suggest that dogs and cats do not pose a serious *Cryptosporidium* infection risk to humans in central Thailand. We expected to detect *C. parvum* in the dog samples from cattle farms, due to their proximity to cattle; the prevalence of *Cryptosporidium* in cattle in Thailand has been reported to be 9.5–13.0% [22, 35]. However, no *C. parvum* was found in the dog samples from the cattle farms. The reason for this negative result is unknown; specific *C. parvum* subtypes may only infect dogs. Further epidemiological and subtype studies of *C. parvum* in dogs are required.

**Table 3.** Country and *Cryptosporidium* species for dogs and cats, from published records.

Animal	Country	*Cryptosporidium* species (number of identified samples)	References
Dog			
	Australia	*C. canis* (4)	[39]
	Italy	*C. canis* (1) and *C. parvum* (7)	[12]
	Thailand	*C. canis* (2)	This study
	USA	*C. muris* (6)	[26]
Cat			
	Australia	*C. felis* (18)	[39]
	Colombia	*C. felis* (5) and *C. muris* (1)	[45]
	Thailand	*C. felis* (2)	This study
	USA	*C. felis* (12)	[4]

Currently, no molecular tools are available to evaluate the subtype characteristics of *C. canis* or *C. felis*; therefore, the host specificity of these species has not been fully explored. In contrast, subtype analyses are available for *C. parvum*, and the variety of host specificity has been reported; some subtypes (IIa, IId) are zoonotic, while another (IIc) is anthroponotic [58]. Unfortunately, *C. parvum* subtypes have not been reported for dogs.

This study had some limitations. First, the sample size was not large enough to analyze the species/genotype characteristics in the region fully. Second, the birds’ fecal samples were combined into sets (10 samples = 1 set for pigeons, 70 samples = 1 set for seagulls), so that the actual prevalence could not be precisely determined. In view of the above findings, further studies are necessary as the number of seagulls, pigeons, dogs, and cats is very large and their proximity to humans makes contamination very likely.

In conclusion, we identified *C. meleagridis* in pigeons, *Cryptosporidium* avian genotype III in seagulls, *C. canis* in dogs, and *C. felis* in cats. Our study indicates that further molecular epidemiological investigations of *Cryptosporidium* in animals, especially pigeons, are necessary to evaluate their possible role as reservoir hosts, and the potential risk they pose to humans.
